# Osmotic and Gibbs–Donnan equilibrium for ions and neutral solutes

**DOI:** 10.1038/s41598-023-47592-w

**Published:** 2023-11-29

**Authors:** Jacek Waniewski

**Affiliations:** Nalecz Institute of Biocybernetics and Biomedical Engineering, Polish Academy of Sciences, Ks. Trojdena 4, 02-109 Warsaw, Poland

**Keywords:** Biophysics, Biotechnology, Cell biology, Physiology, Nephrology, Engineering, Physics

## Abstract

The general set of equations for the equilibrium of two solutions with a mixture of non-permeating and permeating ions and neutral solutes at each side of a permselective membrane is formulated using the principles of electroneutrality and mass conservation law for each solution, and equilibrium conditions: equality of electrochemical potentials at both sides of the membrane for each permeating solution component. There is at least one permeating neutral chemical species (solvent) in the system. The theory is in general valid for non-ideal solutions. The generalized Gibbs–Donnan (G–D) equilibrium coefficients depend on activities/fractions of all species at one side of the membrane, and charges of ions and partial molar volumes of all species. The equilibrium osmotic pressure across the membrane is also provided by the theory and can be calculated using the ratio of activities (or equivalently the G–D factor) of any permeating neutral solute (including solvent) or the ratios of activities (or equivalently the G–D factors) of any two permeating ions.

## Introduction

The Gibbs–Donnan equilibrium across a permselective membrane is frequently analyzed as equilibrium for solutions that include different ions, and some of them can penetrate across the membrane while the other ions cannot cross the membrane ^1–4^. The presence of neutral (permeating and non-permeating) solutes and (neutral) solvent and their equilibrium is typically ignored. However, the equilibrium conditions in general solutions must involve all solutes and solvent, and, in the presence of non-permeating solutes, not only hydrostatic but also osmotic pressure needs to be considered ^5–8^. Such generalized theory may provide some corrections to the available results and suggest the description of new phenomena. For example, it appears from our theory that Gibbs–Donnan (G–D) effect can be observed also for neutral solutes, although it is much weaker for typical physiological concentrations. Furthermore, the presence of neutral solutes in the mixture of ions may influence the Gibbs–Donnan factors for ions.

The theory is based first of all on the relationships between solute activity and the electrostatic potential and hydrostatic pressure of the fluid (i.e., on the electrochemical potential). The electrostatic neutrality of the solution is the second component of the theory, while the mass balance of all species (including solvent) is the third component. The second and third components involve the molar fractions of the species. To solve this problem we need a relationship between species activity and molar fraction—this is frequently assumed to be described by the activity coefficients ^3,7,9^.

The standard theory of Gibbs–Donnan factors includes their dependence on the charge of the involved ions ^1–4^. In general however, as we show in this study, the partial volumes of all species in the solution are also involved in the conditions for equilibrium, Section "The Gibbs–Donnan theory for solution of neutral solutes and ions solution". This is also true if the solution contains only charged species, Section "Generalized Gibbs–Donnan factors". The equilibrium conditions for neutral species depend directly on their partial volumes only, Section "The Gibbs–Donnan theory for multi-ion solutions". The osmotic pressure of ideal solutions is related to the ratio of solvent (or, equivalently, any permeating neutral solute) molar fractions at both sides of the membrane. For non-ideal solutions the equilibrium conditions (i.e., generalized Gibbs–Donnan factors and osmotic pressure) depend also on the activity coefficients.

## Theoretical derivations

### The Gibbs–Donnan theory for solution of neutral solutes and ions solution

Let us consider two compartments, denoted as 1 and 2, separated by a selectively permeable membrane. Each compartment contains a solution with multiple charged species, multiple neutral species (including solvent), some of which the membrane is permeable to (permeating ions and neutral solutes) and some other to which the membrane is not permeable (non-permeating charged and neutral solutes). The system is considered isothermal and the solutions incompressible.

We also assume that all considered permeating solutes are present only in free state, i.e., that they do not form any chemical compounds (pairs or complexes) with other solutes. For the description of a general case with the solutes present in various chemical forms, see Appendix B in the Supplementary material of reference ^4^. We also assume that none of the permeating solutes are bound to non-permeating species. Moreover, we assume that the solutes do not adsorb on the membrane; this assumption is always fulfilled if the membrane may be considered infinitesimally thin.

The solution in each compartment contains n permeating ions with charge number $z_{i}$ and molar fraction $X_{{\text{i}}}$, $i$ = 1, 2,…*n*. The non-permeating charged (npc) species in each solution are described by $Z_{npc} X_{npc}$, where $Z_{npc} = \sum\limits_{\gamma = 1}^{u} {z_{\gamma } X_{\gamma } } /X_{npc}$, where $u$ is the number of different non-permeating ions, is their average (molar fraction-weighted) charge number and $X_{npc} = \sum\limits_{\gamma = 1}^{u} {X_{\gamma } }$ is their total molar fraction (fixed in each compartment).

The solution in each compartment contains m permeating neutral solutes with molar fraction $X_{\beta }$, $\beta$ = *n* + 1, *n* + 2,…*n* + *m*. The non-permeating neutral (npn) species in each solution are described by their total molar fraction $X_{npn}$ (fixed in each compartment). We assume that in the system there is at least one neutral permeating species—solvent, which molar fraction is denoted $X_{w}$.

The electroneutrality of the solution in each compartment requires:

1 $$\sum\limits_{i = 1}^{n} {z_{i} } X_{i,\alpha } + Z_{npc,\alpha } X_{npc,\alpha } = 0$$

where $\alpha = 1,2$ for compartments 1 and 2, respectively, and, from the definition of molar fractions (c.f. Appendix):

2 $$\sum\limits_{i = 1}^{n} {X_{i,\alpha } } + \sum\limits_{\beta = n + 11}^{n + m} {X_{\beta ,\alpha } } + X_{npc,\alpha } + X_{npn,\alpha } + X_{w,\alpha } = 1$$

Equation (2) codes the physical principle of mass conservation, i.e., the overall molar mass of the solution in each compartment separately is the sum of molar masses of all its constituents in this compartment.

The equilibrium of two multi-solute solutions separated by a permselective membrane requires the intercompartmental equilibration of the electrochemical potentials of each permeating ion and chemical potentials of each permeating neutral solute as well as the equilibration of the chemical potential of the solvent in both compartments. Let us also assume that there is no electrochemical gradient across the membrane caused by mechanisms other than the G-D effect discussed here (e.g., no active transport of ions across biological cell membranes). However, we assume that there is a chemical gradient caused by a hydrostatic pressure difference across the membrane due to effect of non-permeating ions, that is osmotic pressure ^2,3^. Thus, we consider here the electro-diffusive-osmotic equilibrium of all components of the solution.

At the state of equilibrium, the activities of permeating ion *i*, $a_{i}$, in two compartments separated by a permselective membrane are related to the potential $\Delta \Phi$ and pressure $\Delta P$ difference across the membrane as follows ^3,7^, c.f. Appendix:

3 $$\frac{{a_{j,2} }}{{a_{j,1} }} = \exp \left( { - \frac{{z_{j} F}}{RT}\Delta \Phi - \frac{{\overline{V}_{j} }}{RT}\Delta P} \right)$$

where *R* is the ideal gas constant, *T* is the absolute temperature of the mixture, F is the Faraday constant, $\overline{V}_{i}$ is the partial molar volume of solute *i*, $\Delta \Phi = \Phi_{2} - \Phi_{1}$, and $\Delta P = P_{2} - P_{1}$. Thus:

Selecting ion 1 as the reference permeating ion (note that any ion can be selected as a reference ion) and solvent w as the reference permeating neutral solute (note that any permeating neutral species can be selected as the reference neutral solute) one gets:

4 $$\frac{{a_{j,2} }}{{a_{j,1} }} = \exp \left( { - \frac{{z_{1} F}}{RT}\Delta \Phi } \right)^{{z_{j} /z_{1} }} \exp \left( { - \frac{{\overline{V}_{w} }}{RT}\Delta P} \right)^{{\overline{V}_{j} /\overline{V}_{w} }}$$

At the state of equilibrium, the activities of permeating neutral solute *β*, $a_{\beta }$, (including solvent activity $a_{w}$) in two compartments separated by a permselective membrane are related to the pressure difference $\Delta P$ across the membrane as follows ^3,7^, c.f. Appendix:

5 $$\frac{{a_{\beta ,2} }}{{a_{\beta ,1} }} = \exp \left( { - \frac{{\overline{V}_{\beta } }}{RT}\Delta P} \right)$$

where $\overline{V}_{\beta }$ is the partial molar volume of solute $\beta$. Selecting solvent $w$ as a reference species one gets:

6 $$\frac{{a_{\beta ,2} }}{{a_{\beta ,1} }} = \exp \left( { - \frac{{\overline{V}_{w} }}{RT}\Delta P} \right)^{{\overline{V}_{\beta } /\overline{V}_{w} }}$$

Let us denote:

7 $$x = \exp \left( { - \frac{{z_{1} F}}{RT}\Delta \Phi } \right)$$

8 $$y = \exp \left( { - \frac{{\overline{V}_{w} }}{RT}\Delta P} \right)$$

Then, from Eqs. (4) and (6):

9 $$a_{i,2} = a_{i,1} x^{{z_{i} /z_{1} }} y^{{\overline{V}_{i} /\overline{V}_{w} }}$$

10 $$a_{\beta ,2} = a_{\beta ,1} y^{{\overline{V}_{\beta } /\overline{V}_{w} }}$$

and in particular:

11 $$a_{w,2} = a_{w,1} y$$

From now on, we assume that each species activity may be described as equal to its molar fraction multiplied by an activity coefficient, $$a_i = f_i X_i$$, where $$f_{i}$$ is the activity coefficient that may in general be a function of the composition of the solution, in particular of $$ X_i$$. Then, from Eqs. (9)–(11):

12 $$X_{i,2} = K_{i,21} X_{i,1} x^{{z_{i} /z_{1} }} y^{{\overline{V}_{i} /\overline{V}_{w} }}$$

13 $$X_{\beta ,2} = K_{\beta ,21} X_{\beta ,1} y^{{\overline{V}_{\beta } /\overline{V}_{w} }}$$

14 $$X_{w,2} = K_{w,21} X_{w,1} y$$

with

15 $$K_{j,21} = \frac{{f_{j,1} }}{{f_{j,2} }}$$

where j represents any of the subscripts *i*, $\beta$ or w.

Let us also assume that all molar fractions of permeating species in compartment 1 are known and we want to calculate the respective equilibrium molar fractions of permeating species in compartment 2 together with the equilibrium differences in electrostatic potential and hydrostatic pressure between the compartments.

Using Eqs. (12)–(15) and mass balance for compartment 2, Eq. (2) for $\alpha = 2$, one gets:

16 $$\sum\limits_{i = 1}^{n} {K_{i,21} X_{i,1} } x^{{z_{i} /z_{1} }} y^{{\overline{V}_{i} /\overline{V}_{w} }} + \sum\limits_{\beta = n + 1}^{n + m} {K_{\beta ,21} X_{\beta ,1} } y^{{\overline{V}_{\beta } /\overline{V}_{w} }} + X_{npc,2} + X_{npn,2} + K_{w,21} X_{w,1} y = 1$$

From the condition of electrostatic neutrality in compartment 2, Eq. (1) for $\alpha = 2$:

17 $$\sum\limits_{i = 1}^{n} {z_{i} K_{i,21} X_{i,1} x^{{z_{i} /z_{1} }} y^{{\overline{V}_{i} /\overline{V}_{w} }} } + Z_{npc,2} X_{npc,2} = 0$$

In general, the system of two Eqs. (16) and (17) needs to be solved numerically for $x$ and $y$.

If the composition of fluids at both sides of the membrane is the same, i.e., if molar fractions for all species are the same in compartments 1 and 2, then the activity coefficients $f$ are also the same and therefore all the parameters $K$ are equal to one [see Eq. (15)], and Eqs. (16) and (17) are automatically valid for this case. One can however obtain more general conditions for equality of molar fractions at both sides of the membrane under additional assumptions on the involved solutions. For example, if the solutions are ideal, i.e., all the coefficients $f$ and parameters $K$ are equal to one, then variables $x$ and $y$ are equal to one if $X_{npc,2} + X_{npn,2} = X_{npc,1} + X_{npn,1}$, $Z_{npc,2} X_{npc,2} = Z_{npc,1} X_{npc,1}$. To prove this, let us write Eqs. (16) and (17) for ideal systems and $x = y = 1$:

18 $$\sum\limits_{i = 1}^{n} {X_{i,1} } + \sum\limits_{\beta = n + 1}^{n + m} {X_{\beta ,1} } + X_{npc,2} + X_{npn,2} + X_{w,1} = 1$$

19 $$\sum\limits_{i = 1}^{n} {z_{i} X_{i,1} } + Z_{npc,2} X_{npc,2} = 0$$

that, with our additional assumption on non-permeating species, are simply our base Eqs. (1) and (2) for $\alpha = 1$.

The mathematical solution $x \, = \, 1$ and ${\text{y }} = \, 1$ in general describes the equilibration of activities of all ions, neutral solutes as well as of electrostatic potential and hydrostatic pressure across the membrane. In particular, these conditions are satisfied if there is no non-permeating ions nor non-permeating neutral solutes, or if the non-permeating species have the same composition at both sides of the membrane. However, for ideal systems, only total molar fraction and total electrostatic equivalent of non-permeating species need to be equal whereas the specific composition of these species may be different at the opposite sides of the membrane.

It is worth to notice that in general we cannot consider the molar fractions of non-permeating solutes in compartment 2, $X_{npc,2}$ and $X_{npn,2}$, as known, because the fractions of other solutes are to be found. Instead, we may assume other measures of non-permeating solutes as known, for example the molar volumetric concentrations, $c_{npc,2}$ and $c_{npn,2}$, that are frequently fixed in experimental studies. Then, using the relationships between $c$ and $X$, see Appendix, one may derive from Eqs. (16) and (17) the following equations for *x* and *y*:

20 $$\sum\limits_{i = 1}^{n} {r_{i} K_{i,21} X_{i,1} } x^{{z_{i} /z_{1} }} y^{{\overline{V}_{i} /\overline{V}_{w} }} + \sum\limits_{\beta = n + 1}^{n + m} {r_{\beta } K_{\beta ,21} X_{\beta ,1} } y^{{\overline{V}_{\beta } /\overline{V}_{w} }} + r_{w} K_{w,21} X_{w,1} y = 1$$

21 $$\begin{aligned} & \sum\limits_{i = 1}^{n} {\left( {z_{i} + s_{i} Z_{npc,2} } \right)K_{i,21} X_{i,1} x^{{z_{i} /z_{1} }} y^{{\overline{V}_{i} /\overline{V}_{w} }} } + \sum\limits_{\beta = n + 1}^{n + m} {s_{\beta } Z_{npc,2} K_{\beta ,21} X_{\beta ,1} } y^{{\overline{V}_{\beta } /\overline{V}_{w} }} \\ & \quad + \;s_{w} Z_{npc,2} K_{w,21} X_{w,1} y = 0 \\ \end{aligned}$$

where

22 $$r_{j} = 1 + \frac{{\left( {c_{npc,2} + c_{npn,2} } \right)\overline{V}_{j} }}{{1 - \left( {c_{npc,2} \overline{V}_{npc} + c_{npn,2} \overline{V}_{npn} } \right)}}$$

23 $$s_{j} = \frac{{c_{npc,2} \overline{V}_{j} }}{{1 - \left( {c_{npc,2} \overline{V}_{npc} + c_{npn,2} \overline{V}_{npn} } \right)}}$$

with subscript $j$ denoting any of subscripts $i$, $\beta$ and $w$ (see Appendix for details of the derivation).

### Generalized Gibbs–Donnan factors

The generalized Gibbs–Donnan (G–D) factor for species $j$ ($j = i,\beta ,w$) according to the extended theory can be defined as:

24 $$DF_{j,21} = \frac{{a_{j,2} }}{{a_{j,1} }}$$

and, for known solutions x and y of Eqs. (20)–(21), it can be calculated as:

25 $$DF_{j,21} = x^{{z_{j} /z_{1} }} y^{{\overline{V}_{j} /\overline{V}_{w} }}$$

Thus, the calculation of $a_{j,2}$ if $a_{j,1}$ is known may be performed for the known G-D factors:

26 $$a_{j,2} = DF_{j,21} a_{j,1}$$

and for calculation of $X_{i,2}$ if $X_{i,1}$ and activity coefficients f are known as:

27 $$X_{j,2} = DF_{j.21} K_{j,21} X_{j,1}$$

In particular, the Gibbs–Donnan factor for neutral solute $\beta$ (the electrostatic neutrality means $z_{j} = 0$ in Eq. (25), c.f. Equation (10), is:

28 $$DF_{\beta ,21} = y^{{\overline{V}_{\beta } /\overline{V}_{w} }}$$

Furthermore, the G-D factor for solvent is:

29 $$DF_{w,21} = y$$

Note also that osmotic transmembrane pressure, that is the hydrostatic pressure that counteracts osmotic pressure of non-permeating species to provide the equilibrium in the system, can be calculated, if $y$ was found as a solution of Eqs. (20)–(21), as:

30 $$\Delta P = - \frac{RT}{{\overline{V}_{w} }}\ln \left( y \right)$$

or, equivalently, using Eq. (29), as:

31 $$\Delta P = - \frac{RT}{{\overline{V}_{w} }}\ln \left( {DF_{w,21} } \right) = - \frac{RT}{{\overline{V}_{w} }}\ln \left( {\frac{{a_{w,2} }}{{a_{w,1} }}} \right)$$

If other permeating neutral solutes are present in the system, then their activities (or G-D factor) can also be used to calculate osmotic pressure in the system:

32 $$\Delta P = - \frac{RT}{{\overline{V}_{\beta } }}\ln \left( {DF_{\beta ,21} } \right) = - \frac{RT}{{\overline{V}_{\beta } }}\ln \left( {\frac{{a_{\beta ,2} }}{{a_{\beta ,1} }}} \right)$$

To calculate osmotic pressure using G-D factors (or equilibrium activities) for permeating ions, one should use two such ions, c.f. Eq. (25):

33 $$\Delta P = - RT\frac{{z_{k} \ln \left( {DF_{i,21} } \right) - z_{i} \ln \left( {DF_{k,21} } \right)}}{{z_{k} \overline{V}_{i} - z_{i} \overline{V}_{k} }}$$

if $$z_{k} \overline{V}_{i} - z_{i} \overline{V}_{k} \ne 0$$.

The equilibrium electrostatic potential difference $\Delta \Phi$ can be calculated from Eq. (7), if $x$ was found as a solution of Eqs. (20)–(21), as:

34 $$\Delta \Phi = - \frac{RT}{{z_{1} F}}\ln \left( x \right)$$

Each G–D factor depends on the overall composition of the mixture, i.e. $n$ different permeating ions, their charge numbers $z$, $m$ neutral species, the partial molar volumes $\overline{V}$ of all species (including solvent), their (and solvent) activity coefficients, and molar fractions of all species in compartment 1, and the amount of non-permeating species in compartment 2. In general, the G–D factors depend on the properties of all permeating and non-permeating species.

The activity coefficients need to be known as the functions of molar fractions and other thermodynamic parameters of the system ^3,7,9^. However, the composition of the solution in compartment 2 is unknown, so the coefficients *K*, Eq. (15), are functions of *x* and *y*, and in general this dependence needs to be taken into account while solving Eqs. (20) and (21) for equilibrium conditions. In many cases, when the molar fractions in both compartments are not much different, the ratios *K* of activity coefficients, Eq. (15), that appear in Eqs. (20) and (21) may be assumed to be approximately one, even if the activity coefficients *k* themselves are different from 1. In the following examples we assume the solutions to be ideal, i.e., all the activity coefficients being equal to one. If necessary, those examples may be extended and activity coefficients incorporated into the general equations presented in this section. We assume also the partial molar volumes as constant, whereas for some system they may depend on the composition of the solution and its thermodynamic parameters ^10,11^. Such variability may be incorporated into the theory if the respective functions are known.

### The Gibbs–Donnan theory for multi-ion solutions

Let us assume, as a special case of the general theory, that the solution contains only charged solutes, i.e., $m = 0$, and a neutral solvent $w$. Then Eqs. (16) and (17) are reduced to:

35 $$\sum\limits_{i = 1}^{n} {K_{i,21} X_{i,1} } x^{{z_{i} /z_{1} }} y^{{\overline{V}_{i} /\overline{V}_{w} }} + X_{npc,2} + K_{w,21} X_{w,1} y = 1$$

36 $$\sum\limits_{i = 1}^{n} {z_{i} } K_{i,21} X_{i,1} x^{{z_{i} /z_{1} }} y^{{\overline{V}_{i} /\overline{V}_{w} }} + Z_{np,2} X_{npc,2} = 0$$

In general, nonlinear Eqs. (35) and (36) need to be solved numerically for $x$ and *y*. If only electro-diffusive equilibrium in the system is considered, the standard conditions for Donnan equilibrium are obtained from Eq. (36) for $y = 1$ or, equivalently, $\Delta P = 0$, and then only Eq. (36) needs to be solved for *x*, compare ^4^:

37 $$\sum\limits_{i = 1}^{n} {z_{i} K_{i,21} } X_{i,1} x^{{z_{i} /z_{1} }} + Z_{np,2} X_{npc,2} = 0$$

#### Example 1.

Let’s consider the (ideal) solution of Na^+^ and Cl^-^ in water with negatively charged non-permeating protein (albumin) in compartment 1. The parameters of the solution components are presented in Table 1. In this example we discuss the concentrations in compartment 1 typical for physiological systems, see Table 2; the concentration of albumin in compartment 2 is assumed zero. After recalculation of concentrations in compartment 1 to molar fractions (see Appendix for the recalculation formula), we can solve the equations for *x* and *y*. The Eqs. (35) and (36) can be reduced to:

38 $$x^{2} y^{{\overline{V}_{Na} /\overline{V}_{w} }} X_{Na,1} + y^{{\overline{V}_{Cl} /\overline{V}_{w} }} X_{Cl,1} + x\left( {yX_{w,1} - 1} \right) = 0$$

39 $$X_{Na,1} x^{2} y^{{\overline{V}_{Na} /\overline{V}_{w} }} - y^{{\overline{V}_{Cl} /\overline{V}_{w} }} X_{Cl,1} = 0$$

**Table 1:** Solute parameters for Examples 1–12.

Ion	Charge number	Partial molar volume (L/mmol)	References	Neutral solute	Partial molar volume (L/mmol)	References
Na^+^	+ 1	− 7.5·10^–6^	^12^	Water	18·10^–6^	^10^
Cl^−^	− 1	24.1·10^–6^	^12^	Glucose	112·10^–6^	^13^
Lysozyme	+ 12	0.01	^10,14^	PEG 10,000	0.01	^15,16^
Albumin	− 16	0.05	^10,17^	Dextran 70	0.07	^15,16^

**Table 2:** Concentrations of ions in compartment 1, and Donnan factors and osmotic pressure for Examples 1–5.

Solution component	Example 1	Example 3	Example 4	Example 5
Concentrations, mmol/L				
Na^+^	140	140	140	140
Cl^−^	124	136	60	72
Lysozyme	0	1	0	1
Albumin	1	1	5	5
Donnan factors				
Na^+^	0.941	0.960	0.655	0.716
Cl^−^	1.063	1.042	1.528	1.397
Lysozyme	NA	0.618	NA	0.023
Water	1.000028	1.000025	1.000520	1.000417
Osmotic pressure, mmHg^a^	30.1	26.9	557.2	447.2

^a^Osmotic pressure is calculated as $- \Delta P$.

Thus, from Eq. (39):

40 $$x^{2} = y^{a} \frac{{X_{Cl,1} }}{{X_{Na,1} }}$$

where $a = (\overline{V}_{Cl} - \overline{V}_{Na} )/\overline{V}_{w}$. From Eqs. (38) and (40):

41 $$2y^{{\overline{V}_{Cl} /\overline{V}_{w} }} X_{Na,1} + y^{a/2} \left( {\frac{{X_{Na,1} }}{{X_{Cl,1} }}} \right)^{1/2} \left( {yX_{w,1} - 1} \right) = 0$$

Equation (41) needs to be solved numerically, and for the assumed parameters we get *x* = 0.941 and $y = 1.000028$. Thus $DF_{Na}$ = 0.941, $DF_{Cl}$ = 1.063, and $\Delta P$ = -30.1 mmHg, see Table 2. Note that the Donnan factors are here defined by the ratio of molar fractions. After recalculation of molar fractions to molar volumetric concentrations, see Appendix, one finds that the Donnan factors based on the ratio of volumetric concentrations are the same within the range of digits taken into account in this example. They are also approximately the same as the Donnan factors calculated for $\Delta P$ = 0 in the standard approach ^4^. The concentrations in compartment 2 are $c_{Na,2}$ = $c_{Cl,2}$ = 138.7 mmol/L.

The predicted equilibrium concentrations expressed per volume of water (solvent), see Appendix, are $cw_{Cl,2}$ = $cw_{Na,2}$ = 138.9 mmol/L for compartment 2, and are approximately equal to the respective concentrations per volume of solution in this compartment. The concentrations per water volume in compartment 1 are: $cw_{Na,1}$ = 147.7 mmol/L and $cw_{Cl,1}$ = 130.8 mmol/L.

#### Example 2.

**Figure 1:**
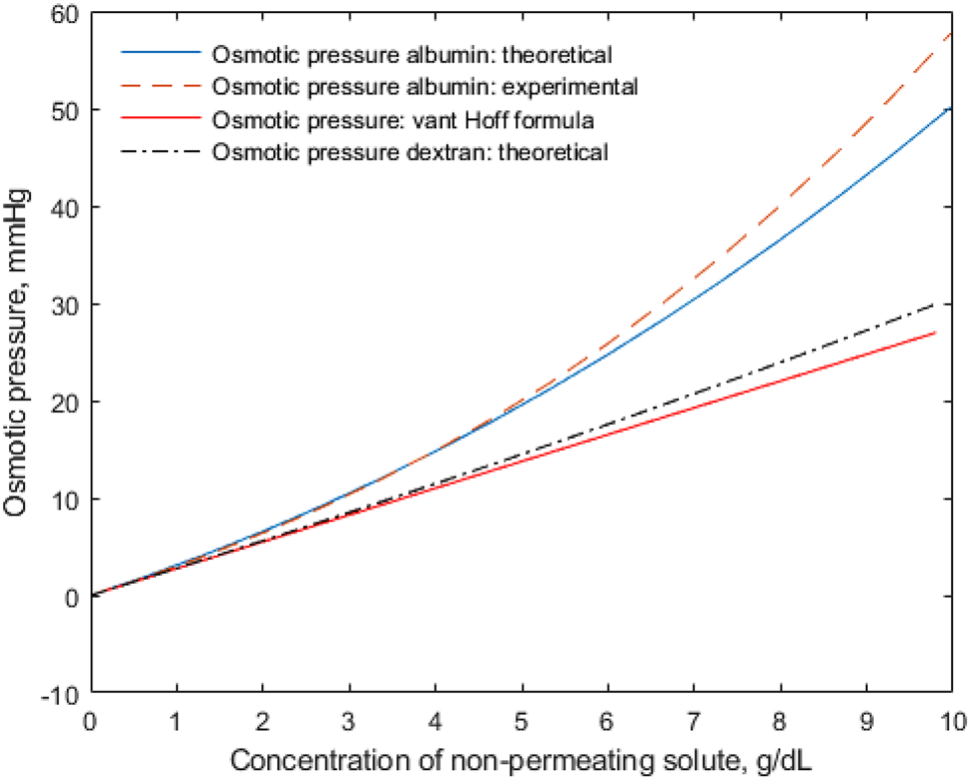
Theoretical prediction of osmotic pressure $- \Delta P$ of albumin in NaCl solution of 150 mmol/L using Eqs. (20) - (21) (continuous line), polynomial curve $- \Delta P = 2.8C_{Alb} + 0.18C_{Alb}^{2} + 0.012C_{{{\text{Alb}}}}^{3}$, $C_{Alb}$ in g/dL, based on the experimental data for pH = 7.4 ^18^ (hatched line), the linear van’t Hoff rule (dotted line), and the theoretical description of osmotic pressure induced by dextran 70 in water solution (dot-hatched line).

Here we compare the predictions from our theory on the change in osmotic pressure with the change of non-permeating solute (albumin) concentration in the solution of NaCl, as in Example 1. The prediction of osmotic pressure of albumin in solution of NaCl of 150 mmol/L at pH = 7.4 compared to experimental data shows that our theory for ideal solution provides reasonably good nonlinear approximation in the physiological range of albumin concentration in human plasma from 3.5 to 5 g/dL ^2,18–20^, see Fig. 1. The calculations were performed assuming, according to the experimental data, Fig. 1, that in compartment 1: $c_{Na,1}$ = 150 mmol/L, $c_{Cl,1}$ = 150 mmol/L, $C_{npc,1}$ = 0 mmol/L, whereas in compartment 2 the concentration of albumin was changed from $C_{npc,2}$ = 0 to $C_{npc,2}$ = 1 mmol/L; Eqs. (20)–(21) were applied.

#### Example 3.

The G-D factors for ions are dependent on two multiplicative terms: (1) generated by the equilibrium Nernst potential and represented by *x*, and (2) generated by osmotic pressure and represented by *y*. The contribution of *y* to G–D coefficients is negligible for the solution described in Example 1 because of low osmotic pressure (i.e., low concentration of non-permeating albumin) and low values of partial molar volumes of Na and Cl. Let us now add to the water solution of albumin, Na and Cl, one more ion: positively charged lysozyme, see Table 1 for the parameters; lysozyme is assumed to permeate across the membrane. Concentrations of solutes in compartment 1 are described in Table 2; for compartment 2: $C_{npc,2}$ = 0 mmol/L. The solution is assumed ideal.

Proceeding as in Example 1 and solving Eqs. (35) and (36) with $n$ = 3, one gets *x* = 0.960 and $y = 1.000025$; the G-D factors and osmotic pressure are provided in Table 2. The concentrations of permeating ions in compartment 2 are: $c_{Na,2}$ = 142.0 mmol/L, $c_{Cl,2}$ = 149.8 mmol/L, $c_{Lys,2}$ = 0.65 mmol/L. Note that G-D factors for small ions (Na and Cl) are slightly closer to 1 and osmotic pressure is lower than for the solution without lysozyme, and that G-D factor for lysozyme is much different from 1. However, the contribution of osmotic pressure to G-D factors is still low, only for lysozyme it increases the factor by around 1.4% ($y^{{\overline{V}_{Lys} /\overline{V}_{w} }}$ = 1.014).

#### Example 4.

To observe the role of osmotic pressure in the lack of equilibration of small ion concentrations, let us consider the solution with high concentration of albumin in compartment 1, see Table 2; for compartment 2: $C_{npc,2}$ = 0 mmol/L. The calculated *x* = 0.655, *y* = 1.000520, and G-D factors and osmotic pressure are presented in Table 2. The concentrations of permeating ions in compartment 2 are: $c_{Na,2} = c_{Cl,2}$ = 122.1 mmol/L.

#### Example 5.

Let us add lysozyme, a small positively charged protein, see Table 1, to the solution discussed in Example 4 at concentration $c_{Lys,1}$ = 1 mmol/L in compartment 1, see Table 2. As the result *x* = 0.716, *y* = 1.000417, and the G–D factors and osmotic pressure are presented in Table 2. Thus, concentrations of permeating ions in compartment 2 are: $c_{Na,2}$ = 135.3 mmol/L, $c_{Cl,2}$ = 135.7 mmol/L, $c_{Lys,2}$ = 0.023 mmol/L. Note that the difference in the concentrations of small ions between compartments and osmotic pressure are considerably lower than in Example 4, at the cost of the difference in the concentrations of lysozyme across the membrane that is increased compared to Example 3. However, the contribution of osmotic pressure to G-D factors is still low, only for lysozyme it increases the G–D factor by around 26.1% ($y^{{\overline{V}_{Lys} /\overline{V}_{w} }}$ = 1.261).

### The Gibbs–Donnan theory for solutions with neutral solutes

Let us now assume that there are only neutral solutes in the solution, i.e., $n = 0$. Then, from Eq. (16):

42 $$\sum\limits_{\beta = 1}^{m} {K_{\beta ,21} X_{\beta ,1} } y^{{\overline{V}_{\beta } /\overline{V}_{w} }} + X_{npn,2} + K_{w,21} X_{w,1} y = 1$$

The osmotic pressure induced by non-permeating dextran 70 (see Table 1) in water solution as function of its concentration, calculated using Eq. (42), is presented in Fig. 1. The nonlinear profile of this curve departs from the van’t Hoff law although the different is not so high as for non-permeating ions represented in Fig. 1 by albumin in saline.

#### Example 6.

Let’s consider the (assumed ideal) solution of glucose in water with dextran 70 as a neutral non-permeating solute in compartment 1, see Table 1 for the parameters of solutes and Table 3 for their concentrations in compartment 1, there is no dextran in compartment 2: $C_{npn,2}$ = 0 mmol/L.

**Table 3:** Concentrations of neutral solutes in compartment 1, and Donnan factors and osmotic pressure for the system of Examples 6–8.

Solution component	Example 6	Example 7	Example 8
Concentrations, mmol/L			
Glucose	6	6	0
PEG 10,000	0	0	6
Dextran 70	1	14	10
Donnan factors			
Glucose	1.000120	1.081	NA
PEG 10,000	NA	NA	1.379
Water	1.000019	1.012575	1.000579
Osmotic pressure, mmHg^a^	20.7	13,378.2	619.9

^a^Osmotic pressure is calculated as $- \Delta P$.

Equation (42) takes the form:

43 $$1 - X_{Glu,1} y^{{\overline{V}_{Glu} /\overline{V}_{w} }} - X_{w,1} y = 0$$

and needs to be solved numerically. For the assumed parameters we get $y$ = 1.000019. Thus $DF_{Glu}$ = 1.000120 and $\Delta P$ = − 20.7 mmHg. The generalized Gibbs–Donnan effect is therefore very weak in this system, but the related osmotic pressure is not negligible in biological systems. Note that the G–D factors calculated for $\Delta P$ = 0, as in the classical approach, are equal to 1.

#### Example 7.

The Gibbs–Donnan effect can be more visible if one assumes higher concentration of non-permeating solute in compartment 1, see Table 3. Then $y$ = 1.012575, the G–D factor and osmotic pressure are presented in Table 3.

#### Example 8.

If we replace glucose by a neutral permeating solute of partial volume similar to the partial volume of lysozyme, for example PEG 10,000, see Table 1, and increase the concentration of dextran, compared to Example 6, see Table 3, then $y$ = 1.000579; the G-D factor for PEG 10,000 and osmotic pressure are presented in Table 3. Without PEG in the system, the osmotic pressure of the pure dextran solution would be 642.2 mmHg. Thus, the disequilibrium in PEG yields lower osmotic pressure compared to the solution of dextran alone.

### The Gibbs–Donnan theory for solutions with a mixture of neutral and charged solutes

The role of charge of non-permeating solute may be seen if one compares Examples 1–8 and Examples 9–12 presented in Table 4. In the latter Examples it is assumed that in the solutions of permeating ions, Na^+^, Cl^−^ and lysozyme (see Table 1 for their parameters) there is also a neutral non-permeating solute, dextran 70. The solutions in Examples 9 and 11 do not contain lysozyme and the predicted Donnan factors for Na^+^ and Cl^−^ are close to one, and osmotic pressure is not much different from that induced by dextran 70 in the solution of neutral permeating solutes, see Example 6 in Table 3, and slightly higher than that predicted by the van’t Hoff law (that is 19.8 mmHg for concentration of 1 mmol/L and 99 mmHg for concentration of 5 mmol/L of non-permeating solute). Adding lysozyme, Examples 10 and 12, has only slight impact on G-D factors of Na^+^ and Cl^−^, and G–D factor for lysozyme is different from one, whereas lysozyme has almost no impact on osmotic pressure, Table 4. In contrast, the charged albumin as a non-permeating solute in the same solutions of ions induces considerably higher osmotic pressure than dextran 70 at the same concentration and that predicted by van’t Hoff law, see Table 2 and Example 2.

**Table 4:** Concentrations of solutes in compartment 1 and Gibbs–Donnan factors and osmotic pressure for Examples 9–12.

Solution component	Example 9	Example 10	Example 11	Example 12
Concentrations, mmol/L				
Na^+^	140	140	140	140
Cl^-^	140	152	140	152
Lysozyme	0	1	0	1
Dextran 70	1	1	5	5
Donnan factors				
Na^+^	1.0000	0.9997	1.000	0.998
Cl−	1.0000	1.0003	1.000	1.002
Lysozyme	NA	1.007	NA	1.046
Water	1.000019	1.000019	1.000120	1.000120
Osmotic pressure, mmHg^a^	20.3	20.3	128.5	128.5

^a^Osmotic pressure is calculated as $- \Delta P$.

## Discussion

The effect of non-permeating ions on the equilibrium concentrations of permeating ions at the opposite sides of the membrane is well known for long time and easily observable in experimental conditions and real biological and physiological systems ^2,3,7^. Its explanation is based on the assumption of the overall neutrality of the solutions and the thermodynamic relationship between electrochemical potential and ion activity/concentration and equilibrium (Nernst) electrostatic potential ^3,7^. The general formulation of the standard theory was presented recently ^4^. However it included only ionic species, without taking into account possible presence of neutral solutes nor the solvent that need also to adjust their equilibrium characteristics. The presented here generalized approach is based on two physical principles, the electroneutrality of the solutions at both sides of the membrane and the conservation of mass, i.e., the total mass is the sum of masses of mixture components, or, equivalently, that molar fractions of all components sum up to one, Eqs. (1) and (2), and the equilibrium conditions: equality of electrochemical potentials at both sides of the membrane for each solution component, Eq. (3). Now, besides the charge of ions, the partial molar volume of each component needs to be taken into account. The theory allows for the calculation of the ratios of permeating solutes activities/molar fractions/concentrations at both sides of the membrane, i.e., the generalized Gibbs–Donnan factors, and the difference of hydrostatic pressure necessary to equilibrate osmotic pressure of non-permeating species as well as the equilibrium electrostatic potential, Section "Generalized Gibbs–Donnan factors"

The consideration of all types of species present in the solutions (permeating and non-permeating, charged and neutral) provides generalization of the classical Gibbs–Donnan formulas ^3,4,7^. Osmotic pressure in the equilibrated system has an impact on the Gibbs–Donnan factors for ions, Eq. (25), as well as defines the G-D factors for neutral solutes, Eq. (28). These corrections are typically very low in biological systems, see Examples, and this observation provides the theoretical foundation for the common practice in reducing the considerations for biological systems to charged solutes only ^2–4,7^. The general theory, as presented here, is however necessary to check to what extent this simplification is practical for other systems with higher concentrations of solutes.

On the other hand, Gibbs–Donnan factors are directly related to osmotic pressure of the solution that is not negligible in many applications. This observation has been previously reported for solvent only, while our results extend it to all permeating neutral solutes and any pair of permeating ions, Section "Generalized Gibbs–Donnan factors".

The non-ideal solutions need additional consideration because some important parameters as the activity coefficients k and partial volumes $\overline{V}$ are in general functions of the composition of the solutions and these functions, mostly nonlinear functions of molar fractions or concentrations, have to be incorporated into the basic Eqs. (20) and (21) before the equations are solved. Such general approach may be difficult to realize and frequently different approximations are proposed for the description of these parameters ^21–26^. Our examples are restricted to ideal systems that are often assumed as approximately valid and used with relatively good results in biological applications ^2–4,7^.

In our description of the theory one neutral solute is selected as “solvent” and this approach facilitates practical applications if this selection is obvious, as for example for diluted solutions. However there is no need for such a selection from the theoretical point of view and any neutral solute can be selected as “solvent”, which means here a reference neutral solute. Similarly, we select one permeating ion as a reference species for other ions. However, this selection is also arbitrary and aimed on facilitating the notation—any permeating ion can be selected as a reference species for charged solutes.

The general scheme for experimental tests of the presented theory is simple: to measure the concentrations at both sides of the membrane at equilibrium together with osmotic pressure and electrostatic potential, and compare to the theoretical predictions if the solutes’ parameters (including activity coefficients) are known. Nevertheless, the G-D factors for solutes with middle molecular mass (as, for example, lysozyme and PEG) has not been measured, although new membranes, so called medium cut off (MCO) membranes, which tend to separate medium from large (as albumin) proteins were recently designed ^27,28^. In the case of very high osmotic pressures some precautions, as degasification of water and the application of a solid membrane, are warranted ^29^. However, the measured G-D factor, electrostatic potential and osmotic pressure may be used for estimation of some of the solute’s parameters, as for example partial molar volume, see Eqs. (31)–(33). In such applications the problem of overfitting may arise, so some caution is necessary.

Our considerations are based on equilibrium thermodynamics and it was not our aim to extend them to linear non-equilibrium thermodynamics, where osmotic pressure and G-D factors have their applications ^3,30^. There was also substantial effort to provide mechanistic interpretation to different concepts from equilibrium thermodynamics, as for example equilibrium osmotic pressure ^31,32^.

In summary, the general theory of thermodynamic equilibrium for solutions separated by semi-permeable membrane in the presence of non-permeating solutes predicts the lack of full equilibration for each permeating solutes as well as the difference in electrostatic potential and osmotic pressure across the membrane. There are two reasons for this phenomenon: solute charge and its steric characteristics (partial volume). For any number of solutes the mathematical problem can be reduced to two algebraic equations for two variables. The Gibbs–Donnan effect due to electrostatic interactions can be observed for ions at physiological concentrations, whereas the impact of steric factor is of importance for solutes with high partial volume and at high osmotic pressure. The new aspects of the presented theory include:

1. If solution separated by a permselective membrane contain non-permeating solutes with different activities/concentrations at opposite sides of the membrane, i.e. there exists osmotic pressure across the membrane, then the activities/concentrations of all permeating solutes are different at opposite sides of the membrane.
2. The ratio of solute activities/concentrations at opposite sides of the membranes, the generalized Gibbs–Donnan factors, depend on osmotic pressure and partial molecular volume of the solute.
3. For permeating neutral solutes, the G–D factors depend only on osmotic pressure and partial molecular volume; for neutral solutes with low partial molecular volume and for low osmotic pressure (as physiological pressures), the G–D factors are close to unity.
4. For permeating ions, the G–D factors depend also, beside osmotic pressure, on electrostatic potential across the membrane and solute charge, as in the classical theory of G–D factors; thus, the presented here theory reduces to the classical one for sufficiently small osmotic pressures.
5. The prediction of the change in osmotic pressure with the change of non-permeating solute concentration is a nonlinear function different from the approximation of van’t Hoff law, and for physiological concentrations of albumin close to the experimental curve.

## Data Availability

All data generated or analysed during this study are included in this published article.

## Appendix

### General background

Electrochemical potential $\mu_{i}$ of a species *i* in the mixture of species:

44 $$\mu_{i} = \mu_{i0} \left( T \right) + RT\ln \left( {a_{i} } \right) + z_{i} F\Phi + \overline{V}_{i} P$$

where $a_{i}$ is the activity of species *i*, $\overline{V}_{i}$ is its partial molar volume, $z_{i}$ is its charge number, $\Phi$ is the electrostatic potential, $P$ is the hydrostatic pressure, *R* is the perfect gas constant, *F* is the Faraday constant, and *T* is the absolute temperature of the mixture. Some of the species may be neutral, i.e. have the charge number *z* = 0.

For the species activity we assume that $a_{i} = f_{i} X_{i}$, where $f_{i}$ is the activity coefficient and $X_{i} = n_{i} /n$ is the molar fraction of species *i* with the number of molecules $n_{i}$, and $n$ is the total number of molecules in the mixture.

Let’s note that the mixture volume $V$ may be presented as ^3^:

45 $$V = \sum\limits_{k = 0}^{N} {n_{k} \overline{V}_{k} }$$

and select the counting of solution components with the solvent as species $k = 0$, and denote it also by subscript *w*, while the other species will be called solutes and denoted with indexes 1, 2…, *N*.

Using the definition of molar fraction $X_{i}$, molar concentration $c_{i} = n_{i} /V$ and Eq. (45) one gets:

46 $$X_{i} = \frac{{n_{i} }}{{\sum\limits_{k = 0}^{N} {n_{k} } }} = \frac{{c_{i} }}{{\sum\limits_{k = 0}^{N} {c_{k} } }} = \frac{{c_{i} }}{{c_{w} + \sum\limits_{k = 1}^{N} {c_{k} } }} = \frac{{c_{i} \overline{V}_{w} }}{{1 + \sum\limits_{k = 1}^{N} {c_{k} \left( {\overline{V}_{w} - \overline{V}_{k} } \right)} }}$$

47 $$c_{i} = \frac{{n_{i} }}{V} = \frac{{X_{i} }}{{X_{w} \overline{V}_{w} + \sum\limits_{k = 1}^{N} {X_{k} \overline{V}_{k} } }} = \frac{{X_{i} }}{{\overline{V}_{w} - \sum\limits_{k = 1}^{N} {X_{k} \left( {\overline{V}_{w} - \overline{V}_{k} } \right)} }}$$

For solute concentration expressed per solvent volume, $cw_{i} = n_{i} /\left( {n_{w} \overline{V}_{w} } \right)$, we have:

48 $$cw_{i} = \frac{{X_{i} }}{{X_{w} \overline{V}_{w} }}$$

and

49 $$X_{i} = \frac{{cw_{i} }}{{\sum\limits_{k = 0}^{N} {cw_{k} } }}$$

### The case of $X_{npc,2} + X_{npn,2} \ne 0$

To derive Eqs. (20) and (21) let us start from a useful formula for any of the species:

50 $$X_{i} = \frac{{n_{i} }}{n} = \frac{{n_{i} }}{V}\frac{V}{n} = c_{i} \frac{{\sum\limits_{k = 0}^{N} {n_{k} \overline{V}_{k} } }}{n} = c_{i} \sum\limits_{k = 0}^{N} {X_{k} \overline{V}_{k} }$$

where the definitions of *X* and *c* are applied together with the formula (45) for *V*. Now:

51 $$X_{npc,2} \overline{V}_{npc} + X_{npn,2} \overline{V}_{npn} = \left( {c_{npc,2} \overline{V}_{npc} + c_{npn,2} \overline{V}_{npn} } \right)\left( {X_{npc,2} \overline{V}_{npc} + X_{npn,2} \overline{V}_{npn} + \sum\limits_{k \ne npc,npn}^{{}} {X_{k} \overline{V}_{k} } } \right)$$

52 $$X_{npc,2} \overline{V}_{npc} + X_{npn,2} \overline{V}_{npn} = \frac{{\left( {c_{npc,2} \overline{V}_{npc} + c_{npn,2} \overline{V}_{npn} } \right)}}{{1 - \left( {c_{npc,2} \overline{V}_{npc} + c_{npn,2} \overline{V}_{npn} } \right)}}\sum\limits_{k \ne npc,npn}^{{}} {X_{k} \overline{V}_{k} }$$

and the summation is carried out over all permeating species.

Applying formula (50) to $i = npc$ one gets:

53 $$X_{npc,2} = c_{npc,2} \left( {X_{npc,2} \overline{V}_{npc} + X_{npn,2} \overline{V}_{npn} + \sum\limits_{k \ne npc,npn}^{{}} {X_{k} \overline{V}_{k} } } \right)$$

and, using Eq. (52):

54 $$X_{npc,2} = \frac{{c_{npc,2} }}{{1 - \left( {c_{npc,2} \overline{V}_{npc} + c_{npn,2} \overline{V}_{npn} } \right)}}\sum\limits_{k \ne npc,npn}^{{}} {X_{k} \overline{V}_{k} }$$

Proceeding in the same way for $i = npn$:

55 $$X_{npn,2} = \frac{{c_{npn,2} }}{{1 - \left( {c_{npc,2} \overline{V}_{npc} + c_{npn,2} \overline{V}_{npn} } \right)}}\sum\limits_{k \ne npc,npn}^{{}} {X_{k} \overline{V}_{k} }$$

Then, inserting Eqs. (54) and (55) into Eqs. (1) and (2), and gathering the terms with the same $X_{k}$ one obtains:

56 $$\sum\limits_{i = 1}^{n} {r_{i} X_{i,2} } + \sum\limits_{\beta = n + 1}^{n + m} {r_{\beta } X_{\beta ,2} } + r_{w} X_{w,1} = 1$$

57 $$\sum\limits_{i = 1}^{n} {\left( {z_{i} + s_{i} Z_{npc,2} } \right)X_{i,2} } + \sum\limits_{\beta = n + 1}^{n + m} {s_{\beta } Z_{npc,2} X_{\beta ,2} } + s_{w} Z_{npc,2} X_{w,2} = 0$$

where coefficients $r_{j}$ and $s_{j}$ are described by Eqs. (22) and (23), respectively.

Finally, the application of formulas (12)–(14) to Eqs. (56) and (57) Eqs. (56) and (57) yields Eqs. (20) and (21).

## References

[1] Donnan FG (1911). Theorie der Membrangleichgewichte und Membranpotentiale bei Vorhandensein von nicht dialysierenden Elektrolyten. Ein Beitrag zur physikalisch-chemischen Physiologie. Zeitschrift für Elektrochemie und angewandte physikalische Chemie.

[2] Guyton A, Hall J (2006). Textbook of Medical Physiology.

[3] Katchalsky A, Curran PF (1967). Nonequilibrium Thermodynamics in Biophysics.

[4] Waniewski J, Pietribiasi M, Pstras L (2021). Calculation of the Gibbs–Donnan factors for multi-ion solutions with non-permeating charge on both sides of a permselective membrane. Sci. Rep..

[5] Nguyen MK, Kurtz I (2004). Determinants of plasma water sodium concentration as reflected in the Edelman equation: Role of osmotic and Gibbs–Donnan equilibrium. Am. J. Physiol.-Renal..

[6] Nguyen MK, Kurtz I (2006). Quantitative interrelationship between Gibbs–Donnan equilibrium, osmolality of body fluid compartments, and plasma water sodium concentration. J. Appl. Physiol..

[7] Katz MA, Bresler EH, Staub NC, Taylor AE (1984). Osmosis. Edema.

[8] Ogston AG, Michel CC (1978). General descriptions of passive transport of neutral solute and solvent through membranes. Prog. Biophys. Mol. Biol..

[9] Rosgen J, Pettitt BM, Bolen DW (2004). Uncovering the basis for nonideal behavior of biological molecules. Biochemistry.

[10] Jirasek F, Garcia EJ, Hackemann E, Galeotti N, Hasse H (2018). Influence of pH and salts on partial molar volume of lysozyme and bovine serum albumin in aqueous solutions. Chem. Eng. Technol..

[11] Millero FJ (1970). Apparent and partial molal volume of aqueous sodium chloride solutions at various temperatures. J. Phys. Chem..

[12] Couture AM, Laidler KJ (1956). The partial molal volumes of ions in aqueous solution. 1. Dependence on charge and radius. Can. J. Chem..

[13] Landge MG, Badade SS, Kendre BV (2014). Partial molar volumes of glucose in aqueous and various alcohol medium at different solutions. Int. J. Chem. Phys. Sci..

[14] Kuehner DE, Engmann J, Fergg F, Wernick M, Blanch HW, Prausnitz JM (1999). Lysozyme net charge and ion binding in concentrated aqueous electrolyte solutions. J. Phys. Chem. B.

[15] Gaube J, Pfennig A, Stumpf M (1993). Vapor-liquid-equilibrium in binary and ternary aqueous-solutions of poly(ethylene glycol) and dextran. J. Chem. Eng. Data.

[16] Nguyen HT, Bouchaudy A, Salmon JB (2022). Microfluidic free interface diffusion: Measurement of diffusion coefficients and evidence of interfacial-driven transport phenomena. Phys. Fluids.

[17] Fogh-Andersen N, Bjerrum PJ, Siggaard-Andersen O (1993). Ionic binding, net charge, and Donnan effect of human serum albumin as a function of pH. Clin. Chem..

[18] Landis EM, Pappenheimer JR, Hamilton WF, Dow P (1963). Exchange of substances through the capillary walls. Handbook of physiology Section 2 Circulation. 2.

[19] Scatchard G, Batchelder AC, Brown A (1946). Preparation and properties of serum and plasma proteins. 6. Osmotic equilibria in solutions of serum albumin and sodium chloride. J. Am. Chem. Soc..

[20] Scatchard G, Batchelder AC, Brown A, Zosa M (1946). Preparation and properties of serum and plasma proteins. 7. Osmotic equilibria in concentrated solutions of serum albumin. J. Am. Chem. Soc..

[21] Van der Weg PB (2009). The electrochemical potential and ionic activity coefficients. A possible correction for Debye–Huckel and Maxwell–Boltzmann equations for dilute electrolyte equilibria. J. Colloid Interface Sci..

[22] Xiao T, Song X (2021). A Systematic way to extend the Debye–Huckel theory beyond dilute electrolyte solutions. J. Phys. Chem. A.

[23] Agena SM, Bogle IDL, Pessoas FLP (1997). An activity coefficient model for proteins. Biotechnol. Bioeng..

[24] Elliott JAW, Prickett RC, Elmoazzen HY, Porter KR, McGann LE (2007). A multisolute osmotic virial equation for solutions of interest in biology. J. Phys. Chem. B.

[25] McBride DW, Rodgers VGJ (2013). Predicting the activity coefficients of free-solvent for concentrated globular protein solutions using independently determined physical parameters. PLoS ONE.

[26] Minton AP (2007). The effective hard particle model provides a simple, robust, and broadly applicable description of nonideal Behavior in concentrated solutions of bovine serum albumin and other nonassociating proteins. J. Pharm. Sci..

[27] Kirsch AH, Lyko R, Nilsson LG, Beck W, Amdahl M, Lechner P (2017). Performance of hemodialysis with novel medium cut-off dialyzers. Nephrol. Dial. Transpl..

[28] Kirsch AH, Rosenkranz AR, Lyko R, Krieter DH (2017). Effects of hemodialysis therapy using dialyzers with medium cut-off membranes on middle molecules. Contrib. Nephrol..

[29] Mauro A (1965). Osmotic flow in a rigid porous membrane. Science.

[30] Waniewski J, Heimburger O, Werynski A, Lindholm B (1992). Aqueous solute concentrations and evaluation of mass transport coefficients in peritoneal dialysis. Nephrol. Dial. Transplant..

[31] Atzberger PJ, Kramer PR (2007). Theoretical framework for microscopic osmotic phenomena. Phys. Rev. E.

[32] Guell DC, Brenner H (1996). Physical mechanism of membrane osmotic phenomena. Ind. Eng. Chem. Res..

